# Genome-Wide Dissection of the MicroRNA Expression Profile in Rice Embryo during Early Stages of Seed Germination

**DOI:** 10.1371/journal.pone.0145424

**Published:** 2015-12-17

**Authors:** Dongli He, Qiong Wang, Kun Wang, Pingfang Yang

**Affiliations:** 1 Key Laboratory of Plant Germplasm Enhancement and Speciality Agriculture, Wuhan Botanical Garden, Chinese Academy of Sciences, Wuhan 430074, China; 2 University of Chinese Academy of Sciences, Beijing 100049, China; 3 College of life science, Wuhan University, Wuhan 430072, China; Nanjing Agricultural University, CHINA

## Abstract

The first 24 hours after imbibition (HAI) is pivotal for rice seed germination, during which embryo cells switch from a quiescent state to a metabolically active state rapidly. MicroRNAs (miRNAs) have increasingly been shown to play important roles in rice development. Nevertheless, limited knowledge about miRNA regulation has been obtained in the early stages of rice seed germination. In this study, the small RNAs (sRNAs) from embryos of 0, 12, and 24 HAI rice seeds were sequenced to investigate the composition and expression patterns of miRNAs. The bioinformatics analysis identified 289 miRNA loci, including 59 known and 230 novel miRNAs, and 35 selected miRNAs were confirmed by stem-loop real-time RT-PCR. Expression analysis revealed that the dry and imbibed seeds have unique miRNA expression patterns compared with other tissues, particularly for the dry seeds. Using three methods, Mireap, psRNATarget and degradome analyses, 1197 potential target genes of identified miRNAs involved in various molecular functions were predicted. Among these target genes, 39 had significantly negative correlations with their corresponding miRNAs as inferred from published transcriptome data, and 6 inversely expressed miRNA-target pairs were confirmed by 5ʹ-RACE assay. Our work provides an inventory of miRNA expression profiles and miRNA-target interactions in rice embryos, and lays a foundation for further studies of miRNA-mediated regulation in initial seed germination.

## Introduction

Rice is not only the staple food for over half of the world population, but also an ideal plant model for monocot seed germination as it has an annotated genome and a well-developed germination system. A series of physiological and biochemical events were involved in seed germination, during which embryo cells transit from a quiescent state to a metabolically active state. In rice, classical studies have defined seed germination as a sequential process following a triphasic model based on water uptake, namely, rapid water uptake in the first 20 hours after imbibition (HAI) (phase I), lag phase for metabolism reactivation (phase II, 20–48 HAI), and radicle emergence for seedling establishment (phase III, after 48 HAI) [1,2]. Albeit no significant change has been observed in phenotypes of the 24 HAI seed, the oxygen uptake was found to be enhanced in parallel with seed wet weight increase [3]. During this period, the pro-mitochondria are converted to the typical cristae-rich mitochondrial structures following recovery of the aerobic respiration for seedling establishment [2,4]. Previous transcriptome study has shown that a significant proportion of transcripts changed in the first 24 HAI, with most changes being observed between 3 and 12 HAI (5,396 up-regulated and 4,935 down-regulated transcripts) [5]. In addition, a phosphorproteome study also suggests that the first 12 HAI is a potentially important signal transduction phase for the initial rice seed germination [6]. Therefore, the first 24 HAI greatly determines the recovery of metabolism activity in rice seed during the germination progress.

Plant microRNAs (miRNAs), generated from stem-loop regions of long primary transcripts by a Dicer-like (DCL) enzyme, are a class of non-coding sRNAs that are abundant and best-characterized with 20-24-nucleotide (nt) length [7]. MicroRNAs can guide the RNA-induced silencing complex (RISC) to cleave mRNA or remodel chromatin through fully or partly complementary base pairing, resulting in mRNA slicing and degradation, translational repression, or chromatin modifications [8,9]. MicroRNAs have been shown to play important roles in plant growth and development, phyto-hormone homeostasis, and stress response. The ancient miRNAs often functionally target the evolutionarily conserved genes. For example, miR167 is involved in ovule and anther development by repressing Auxin Responsive Factor 6 (*ARF6*) [10]; Arabidopsis seeds expressing the miR160-resistant form of *ARF10* are hypersensitive to abscisic acid (ABA) in a dose-dependent manner [11]. Additionally, miR164 and miR319 are important for organ morphogenesis by targeting NAM, ATAF1/2 and CUC2 domain-containing proteins (*NAC*) and TEOSINTE BRANCHED1/CYCLOIDEA/PROLIFERATING (*TCP*) transcription factor families, respectively [12,13]. Some new miRNA-target pairs have been confirmed experimentally, i.e., miR402 targets DEMETER-LIKE protein3 (*DML*3), a putative DNA glycosylase involved in DNA demethylation. Overexpression of miR402 and deficiency of *dml3* accelerates seed germination under stress conditions [14]. As a key regulator, miRNA has been extensively studied in rice for its role in root development, seed maturation, and pollen development [15–18]. The recently released rice dataset of miRBase (V21.0) has documented 332 miRNA families. These identified miRNAs are important for understanding small RNA (sRNA)-mediated gene regulations in rice. However, to date, limited knowledge about miRNA regulation has been obtained in the early stage of rice seed germination.

Rice seed is composed of a dominant starchy endosperm and a genetically vigorous embryo. Proteomic analysis of the dissected endosperms has shown that the biological processes operating in the endosperm are heavily regulated by the embryo during seed germination [19]. Therefore, embryo is an important tissue in seed germination control. In this study, we sequenced the sRNA populations from embryos of 0, 12, and 24 HAI rice seeds using next-generation deep sequencing technology. A series of miRNAs were identified, including both known and novel miRNAs. We also predicted the potential targets for the miRNAs. Real-time RT-PCR and 5ʹ-RACE assay were performed to confirm some deep sequencing and target prediction results. This study provides the unique composition and expression profiles of miRNAs and their potential regulations in the embryo at the early stages of rice seed germination.

## Materials and Methods

### Plant Material and Seed Germination

The de-hulled rice (*Oryza sativa* Nipponbare) seeds were washed three times with distilled water, then placed in 9-cm-diameter plates and imbibed in distilled water at 30°C in a dark biochemical incubator. Fifty seeds were placed in one plate and set up as a biological replicate. Four replicates were prepared for each time-point sample. After 0 h, 12 h, and 24 h of imbibition, embryos were dissected manually and collected for RNA extraction, respectively. For phytohormone treatment, seeds were incubated with 200 μM GA_3_ and 50 mM ABA (Sigma, St. Louis, MO, USA) for 24 h, after which the embryos were collected.

### Small RNA Libraries Construction and Sequencing

Total RNA was extracted with TRIzol Reagent (Invitrogen, Carlsbad, CA, USA) according to the manufacturer’s instructions. Equal amounts (about 25 μg) of total RNA from four replicates of each time-point sample were mixed together for sRNA libraries construction. The sRNA libraries were constructed following the methods described by Lu et al [20]. Briefly, the total RNA was fractionated by 15% denaturing polyacrylamide gel (8 M urea) electrophoresis, and sRNAs in the range of 18–30 nt were excised and purified with a Spin-X cellulose acetate filter (2 mL, 0.45 um; Thermo Fisher, Waltham, MA, USA). After dephosphorylation and sequential ligation of 5ʹ- and 3ʹ- Solexa adaptors, the sRNAs were reverse-transcribed using superscript^TM^II reverse transcription kit (Invitrogen), and then amplified by PCR with phusion High-Fidelity PCR kit (Finnzymes, Espoo, Finland) to produce sRNA sequencing libraries. Next generation sequencing was performed on an Illumina platform (Nextomics Science and Technology Limited, Wuhan, China). All the sequence data have been deposited as a series with the accession number GSE73657 at NCBI’ GEO database (http://www.ncbi.nlm.nih.gov/geo/query/acc.cgi?acc=GSE73657).

### Deep Sequencing and Processing

The raw sequencing data were filtered with FastQC (http://www.bioinformatics.babraham.ac.uk/projects/fastqc/) to delete low quality reads, adapters, contamination, and false sequences (such as reads with poly A/T/C/G). After filtering, sRNAs with size ranging from 18 to 30 nt were collected and mapped to the rice genome (MSU Rice Annotation Release 7.0) using Bowtie software, and then the sequences matching known rice rRNA, tRNA, snRNA, and snoRNA in Rfam11 (https://www.sanger.ac.uk/resources/databases/rfam.html) were discarded. Subsequently, the unique sRNAs were aligned with the data in miRBase 21 (www.mirbase.org) to search for the conserved miRNAs.

*De novo* prediction of novel miRNA was performed by miRDeep2. Genomic sequences with 300 bp length, surrounding the sRNAs (150 nt upstream and 150 nt downstream), were extracted to assess the potential secondary structures of pre-miRNA. The candidates were first filtered with default criteria, and then with the stringent criterion of minimal folding free energy index (MFEI) > 0.85 (MFEI = [(MFE/length of the sRNA sequence) × 100] / (G+C) %) [21].

### Target Prediction of Identified miRNAs

The prediction of putative targets for identified miRNAs was performed with Mireap, PsRNATarget and degradome analyses based on the rice genome (MSU 7.0) [22–24]. For the Mireap software analysis, the following modified parameters were used: 1) no more than four mismatches between miRNA and the target, the G-U base pair was counted as 0.5 mismatches; 2) no more than two adjacent mismatches in the miRNA/target duplex; 3) no adjacent mismatches in positions 2–12 (5ʹ of miRNA); 4) perfect matches in positions 10–11; 5) no more than 2.5 mismatches in positions 1–12; 6) MFE between miRNA and target gene not less than 75% of the MFE for a perfect matching. PsRNATarget prediction software (http://plantgrn.noble.org/psRNATarget) was used with default parameters. Degradome analysis was performed based on GSE18251, GSE17398, and GSE19050 dataset as previously described with p < 0.05 [15,22]. WEGO software (http://wego.genomics.org.cn/) was used for plotting gene ontology annotation results, and Agrigo software (http://bioinfo.cau.edu.cn/agriGO) was used to enrich the GO terms.

### Expression Analysis of miRNAs, Target Genes, and Host Genes

The miRNA expression profiles in multiple tissues were obtained from the published dataset [16]. To compare the miRNA expression in multiple tissues, the Log_2_TPM (Transcripts per million = number of actual reads/total number of clean reads × 1,000,000) value was presented. MeV4.9 was used for hierachical clustering (HCL) and principal component analysis (PCA). The expression patterns of target genes were extracted from the published microarray dataset (GSE43780) [5]. To analyze the expression relevance between a miRNA and its corresponding target gene(s), the TPM value of miRNA or the genechip robust multi-array average (GCRMA) value of the target gene was divided by its maximum to reach abundance between 0 and 1.

### Stem-Loop Reverse Transcription Polymerase Chain Reaction (RT-PCR)

RNA reverse transcription was performed with specific stem-loop primers as previously described [25] (S1 Table). The stem-loop RT-PCR reactions contained 25 ng of RNA samples, 50 nM stem-loop RT primer, 1 × RT buffer, 0.25 mM of each dNTP, 5 U/μL SuperScript II reverse transcriptase (Invitrogen, USA) and 0.25 U/μL RNase inhibitor. The 10 μL reactions were incubated in a 96-well plate for 30 min at 16°C, 30 min at 42°C and 5 min at 85°C, and then held at 4°C in a Bio-Rad professional thermocycler (Bio-Rad, Hercules, California, USA). The real-time PCR amplification program consisted of initial denaturation at 95°C for 3 min, followed by 40 cycles of 95°C for 10 s, 58°C for 15 s, and 72°C for 15 s. Three biological replicates were performed on the CFX96 Real-time PCR system (Bio-Rad, California, USA). The melting curves (65°C to 95°C) were analyzed to check the specificity of PCR products. PCR efficiency was calculated by LinRegPCR (http://www.hartfaalcentrum.nl/; subject: LinRegPCR) considering an ideal value range (1.8–2.05) with correlation R^2^> 0.995 [26]. The relative miRNA expression was calculated using the following formula:

$${\text{Ration=E}}_{\text{target}}{}^{\text{target}\Delta \text{Ct(Control-sample)}}{\text{/ E}}_{\text{ref.}}{}^{\text{ref.}\Delta \text{Ct(Control-sample)}}$$

In which E_target_ represents PCR efficiency of measured miRNA/gene, E_ref_ represents PCR efficiency of reference gene 18S RNA, and ΔCt represents the difference in threshold cycles between the control and sample.

### RNA Ligase-Mediated Rapid-Amplification of 5’-cDNA Ends (5’-RACE)

To investigate the cleavage sites occurring in the targeted mRNAs, 5ʹ-RACE was performed using the FirstChoice^®^ RLM-RACE kit (Ambion, USA) according to the manufacturer’s instructions. Briefly, high quality mRNAs were directly ligated to the 5ʹ-RACE RNA adapter (44 nt), and the ligation reaction was then reverse-transcribed into cDNA with random primers. Two 5ʹ-RACE gene-specific outer and inner primers were used for nested PCR. The PCR products with the anticipated size were cloned and sequenced. The detailed primer sequences are listed in S1 Table.

## Results

### Deep Sequencing of Small RNAs

**Fig 1:**
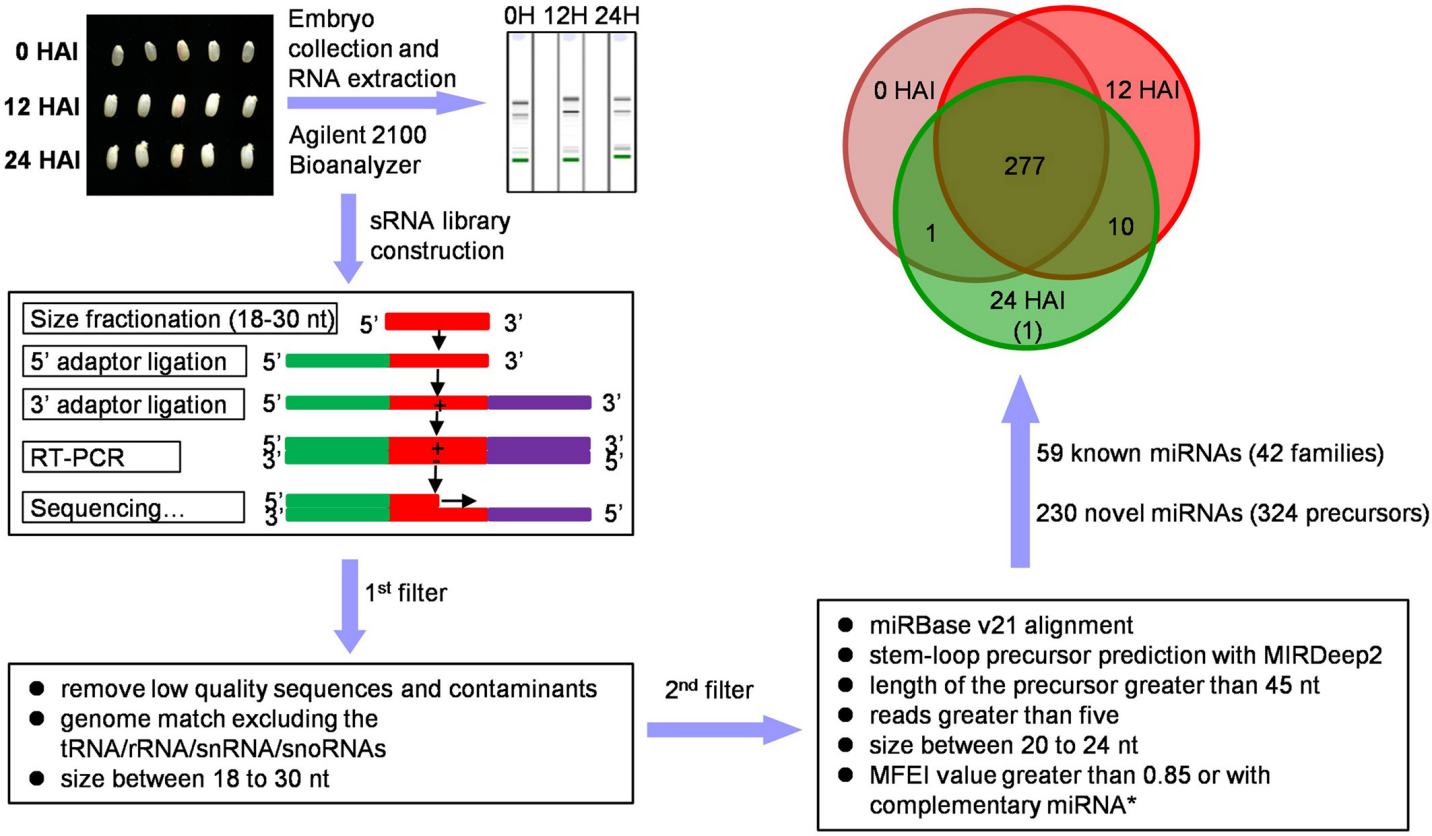
Schematic representation of the miRNA analysis in rice embryos at the early stages of seed germination.

**Fig 2:**
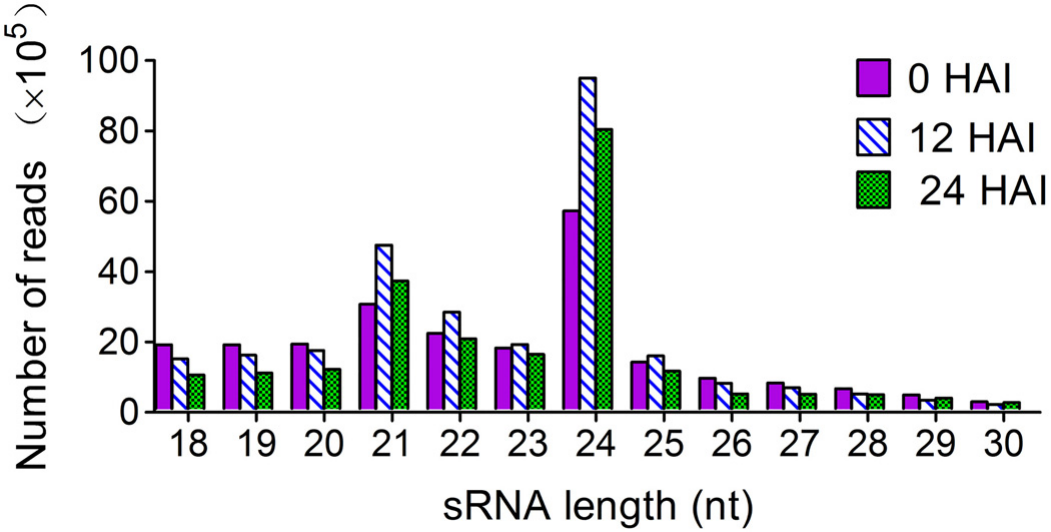
Size distribution of the sRNAs in libraries from 0, 12, and 24 HAI rice seed embryos.

Previous study has shown that large-scale rearrangement of transcripts mediated by RNA synthesis and degradation occurs in the first 24 h of rice seed germination [5]. Using an experimental system similar to that utilized in the previous study [5], the miRNA composition and dynamic expression patterns were investigated in the early stages of rice seed germination. About 200 embryos from 0, 12, and 24 HAI rice seeds were collected for total RNA extraction and sRNA libraries construction, respectively (Fig 1). RNA concentration and integrity were detected using Agilent 2100 Bioanalyzer and Agilent RNA 6000 Nano kit. About 100 μg of high-quality RNA from each sample was used for sRNAs isolation and sequencing. In total, 29230963, 34302280, and 33901503 raw reads with high quality (Q30 > 94%) were obtained for 0, 12, and 24 HAI sRNA libraries, respectively. After removal of low quality sequences and adapter contaminants, 19203513, 22332607, and 17359816 reads for each library were perfectly mapped to the reference rice genome (MSU 7.0) with size ranging from 18 to 30 nt (Table 1). Consistent with the representative size range of DCLs cleavage products [27], most of these sRNAs were 21 or 24 nt in length. The 24-nt sRNAs occurred in a particularly high abundance, accounting for 25.3%, 34.7%, and 37.5% in the three libraries, respectively (Fig 2).

**Table 1:** Statistics for sRNA sequencing from three libraries.

Category	0 HAI		12 HAI		24 HAI	
	Reads^b^	Unique^b^	Reads^b^	Unique^b^	Reads^b^	Unique^b^
high quality raw Reads^a^	29230963 (Q30 = 94.74%)	-	34302280 (Q30 = 95.29%)	-	33901503 (Q30 = 94.58%)	-
sequences of 18–30 nt	22265577(100%)	6596317(100%)	27075645(100%)	10712075(100%)	21236500(100%)	9420781(100%)
mapping to genome	19203513(86.3%)	4856925(73.6%)	22332607(82.5%)	7892704(73.7%)	17359816(81.8%)	7115379(75.5%)
CDS matched	600910(2.7%)	211949(3.2%)	1570839(5.8%)	338298(3.2%)	1322306(6.2%)	243265(2.6%)
**Non-coding RNA**						
total	17642144(100%)	**-** ^c^	19201655(100%)	**-** ^c^	14737124(100%)	**-** ^c^
known miRNA	400047(2.3%)	1724(0.4%)	873690(4.6%)	2506(2.9%)	781907(5.3%)	2314(29.6%)
rRNA	2289143(13.0%)	24748(1.1%)	1388914(7.2%)	23099(1.7%)	747877(5.1%)	19579(2.6%)
tRNA	277627(1.6%)	8121(2.9%)	274039(1.4%)	7349(2.7%)	421275(2.9%)	6878(1.6%)
snoRNA	40642(0.2%)	8790(21.6%)	44927(0.2%)	6001(13.4%)	28636(0.2%)	4755(16.6%)
snRNA	5956(0.34%)	1679(28.2%)	6005(0.32%)	1390(23.1%)	3623(0.25%)	1079(29.8%)
others	14628729(82.9%)	4345729(29.7%)	16614080(86.5%)	7138503(43.0%)	12753806(86.5%)	6489573(50.9%)

^a^: Q30 is the percentage of base with correct recognition rate over than 99.9%.

^b^: the percentage to the 100% reads.

^c^: the percentage to the sequences of corresponding ‘reads’.

The composition of the genome-matched sRNAs of rice seed was complex, which included protein-coding RNA fragments (3.13–7.62%) and other non-coding transcripts. The non-coding sRNAs contained various categories, including known miRNAs (2.09–4.5%), rRNAs (4.31–11.9%), tRNAs (1.45–2.43%), snoRNAs (0.16–0.21%), snRNAs (0.02–0.03%) and unannotated sRNAs, (80.9–84.31%). The unannotated sRNAs were further used to predict the novel miRNAs.

### Identification of Known and Novel miRNAs in Dry and Imbibed Rice Seeds

To scan the known miRNAs, the unique sRNAs were aligned to the rice miRNA datasets in miRBase 21 (http://www.mirbase.org/), allowing up to two mismatches. In the three libraries, 59 known rice miRNAs homologs from 83 precursors corresponding to 42 miRNA families were matched (S2 Table). Of which, 14 families had two or more miRNA members, and the other 28 families contained only one miRNA member. In this study, 17 conserved families were detected in the imbibed rice seeds, most of them were found in relatively high abundance. In particular, miR319, miR168, miR156, miR166, and miR159 were mapped with more than 10,000 reads (S1 Fig). However, some conserved miRNAs, i.e., miR164, mi395, and miR393, were lowly expressed in rice seeds. Nineteen families are known rice-specific miRNAs, and most of them had only one member and were in relatively low abundance. A homolog of ptc-miR6478, previously reported only in *Populus trichocarpa*, was also identified in this study. Moreover, we detected a new precursor with stringent criteria for osa-miR1862 family (named osa-MIR1862d-2). These results show that our sequencing depth was sufficient to reflect the expression profiles of miRNAs during seed imbibition.

For novel miRNAs identification, the 300-bp flanking sequences of unannotated sRNAs were initially extracted, and then subjected to MIRDeep2 to scan potential miRNA precursors with classical stem-loop structures using the default criteria. A total of 382 novel miRNA candidates with length of 18–26 nt processed from 481 putative precursors were obtained from the three libraries. MFEI has been identified to be useful in evaluating genuine miRNAs [21], which depends on the MFE, length and G+C content of the potential precursors. These criteria were used in our investigation of miRNAs in rice seeds. Low MFE is an important characteristic of miRNA precursors. In this study, the miRNA precursor’s MFE values varied from -180.6 to 5.2 kcal·mol^-1^ according to Mfold 3.2 (Figure A in S1 File). In animal, the size of miRNA precursor ranges typically from 70 to 80 nt, but it is more variable in plant.[28]. In rice seeds, the size of miRNA precursors varied from 37 to 268 nt, with most being 51–110 nt(Figure B in S1 File). In addition, the high G+C content can make the pre-miRNA secondary structure more stable and thus difficult to be processed into mature miRNA by the RISC complex [21]. Here, the G+C content ranged from 14.03% to 88% (Figure C in S1 File). With all these factors in consideration, we calculated the MFEI value of all identified miRNA precursors as previously described [21]. Previous research has shown that more than 90% of known plant pre-miRNAs have an MFEI value greater than 0.85 [21]. In this study, the MFEI values ranged from 0.20 to 3.75, with an average of 1.26. To minimize the false positive miRNAs, we further set the novel miRNA criteria as follows: 1) the mature miRNA length ranged from 20 to 24 nt; 2) precursor had a classical fold-back structure and the length was no less than 45 nt; 3) the number of reads was greater than five in at least one library; 4) MFEI value was greater than 0.85 or the complementary miRNA* could be found. Finally, 230 novel miRNAs processed from 324 precursors were obtained (Fig 1). The detailed information was listed in S3 Table. Of these novel miRNAs, 218 were shared by all three libraries. The novel miRNA was denoted with OsmiR plus a serial number, such as OsmiR-1. Different miRNA precursors that produced the same mature sequence were noted with -1, -2, and -3.

**Fig 3:**
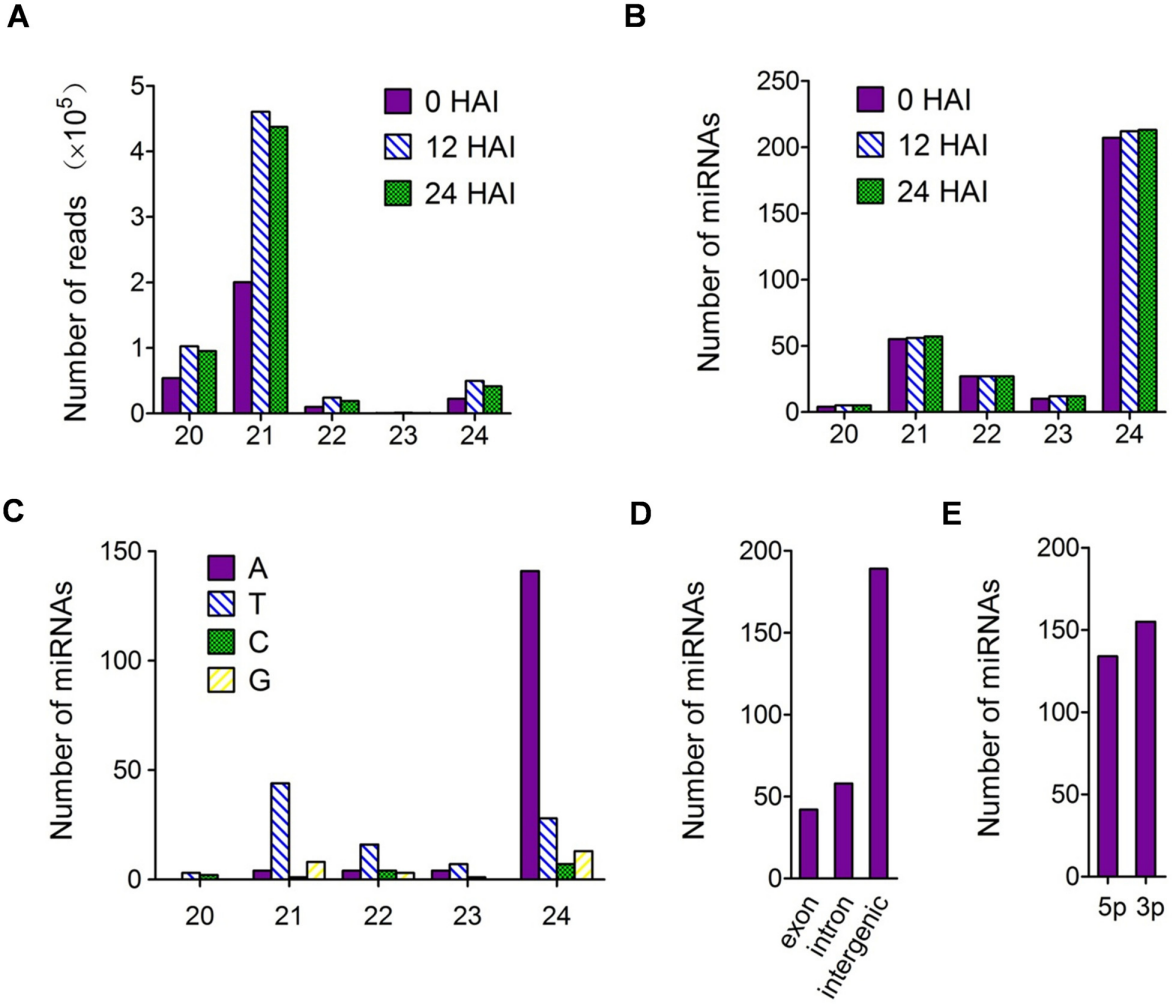
Characteristics of all identified miRNAs in libraries from 0, 12 and 24 HAI rice seed embryos. Distribution of sequencing reads **(A)**, number of miRNAs **(B)** and the first base of miRNAs **(C)**. **(D)** Genome and **(E)** precursor arm locations of all identified miRNAs.

In plants, most of the canonical miRNAs (∼21 nt) are in high abundance [29]. Similarly, in this study, the 21-nt miRNAs occupied an overwhelming majority of the reads (Fig 3A, S2 Table). Moreover, a great number of long miRNAs (24 nt) with low abundance existed in the early stages of rice seed germination (S3 Table and Fig 3B). Specifically, of the 230 novel miRNAs, 176 were 24-nt. The miRNAs function through interacting with AGO proteins, which bind to the miRNA with particular 5ʹ end nucleotide. AGO1 mainly recruits miRNAs that begin with a 5ʹ-U/T; AGO2 prefers to bind sRNAs with 5ʹ -A [30,31]. The first base distribution of all 289 identified miRNAs showed that the first base of 24-nt miRNAs were tended to be A (66.2%), suggesting these miRNAs function with AGO2; while that of the shorter miRNAs were inclined to be U/T (69.3%) (Fig 3C). Genome mapping revealed that most known and novel miRNAs were located in the intergenic regions, and fewer in the intragenic regions (i.e., exon, intron). In addition, the miRNAs were located randomly on the 5p or 3p arm of their precursors (Fig 3D and 3E).

To validate the deep sequencing results, 35 (17 known and 18 novel) miRNAs with sequence reads higher than 100 were randomly selected for stem-loop RT-PCR analysis. All the 35 amplified miRNAs produced a single PCR band with the length of about 60 bp on the gel (The amplified PCR product included the 20–24 bp miRNA sequence, part of the 44 bp stem-loop primer sequence (that is 34 bp) and several primer balance bases) (S2 Fig). The PCR products were cloned and sequenced. All the tested miRNAs sequences were verified to be quite correct to that of the sequencing, demonstrating a high reliability of the deep sequencing results.

### The Expression Patterns of Known and Novel miRNAs During Rice Seed Imbibition

**Fig 4:**
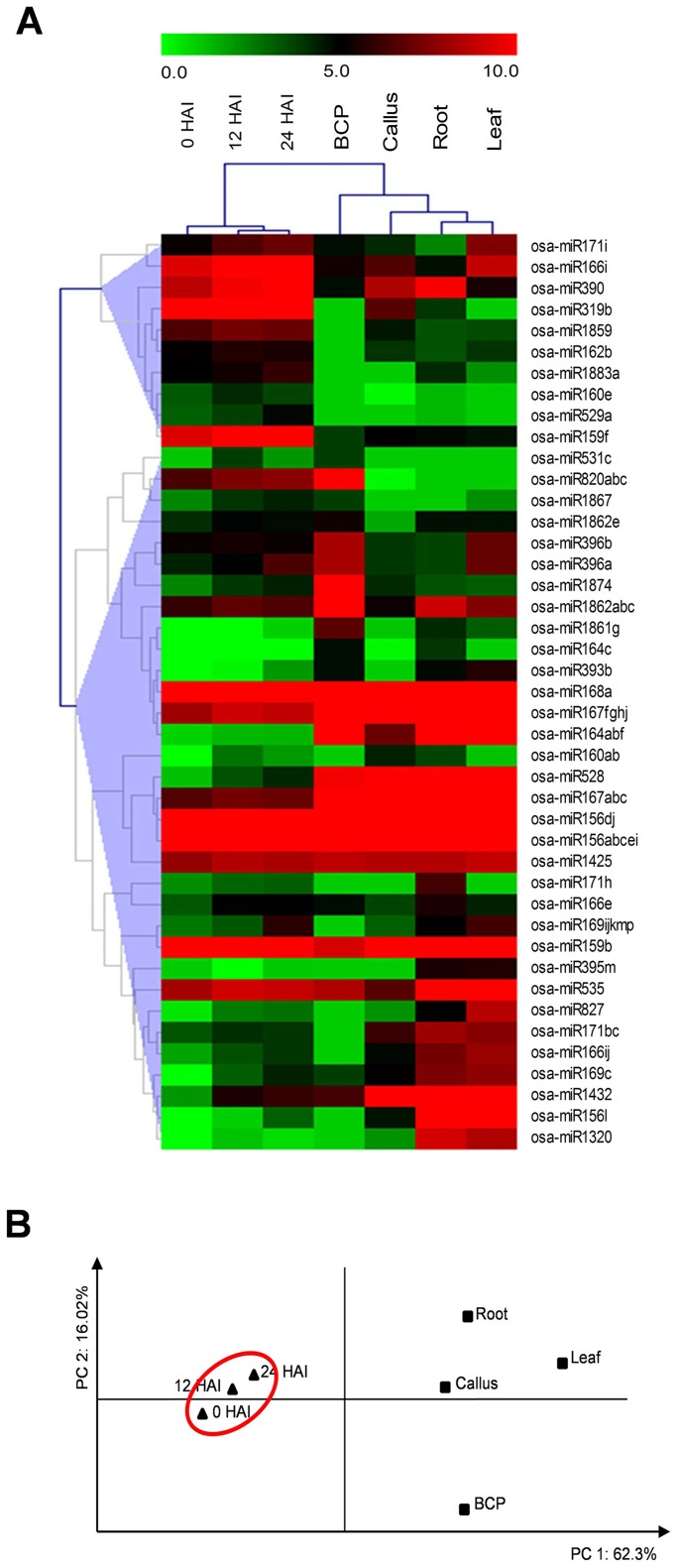
Analyses of tissue-specific expression pattern of known miRNAs. Hierarchical clustering analyses **(A)** and principal component analysis **(B)** of known miRNAs in various tissues. Clustering of known miRNAs based on Log_2_TPM. The bar represents the scale of miRNAs expression levels of miRNAs. The detailed information is listed in Table A in S2 File.

To investigate the tissue expression pattern of the miRNAs, the expression data of known miRNAs in bicellular pollen (BCP), leaf, root and callus of rice were obtained from the previous report (Table A in S2 File) [16]. HCL analysis and PCA were performed with Log_2_TPM value of known miRNAs from BCP, leaf, root, callus, and embryos of 0, 12, and 24 HAI rice seeds. Results indicated that 7 samples were significantly clustered into two clades, and the seed embryo samples were separated from the others (Fig 4).

In total, 286499, 636508, and 592581 miRNA reads were obtained from 0, 12, and 24 HAI libraries, respectively. Although a significantly smaller amount of miRNAs were mapped in the dry seeds (0 HAI) than that in the imbibed seeds (12 HAI and 24 HAI), the amount of genome-mapped sRNAs reads was substantial (Table 1), which implied the existence of a large number of other categories of sRNAs, i.e., siRNAs, in dry seeds. HCL analysis using the LOG_2_TPM value of miRNAs showed that 0 HAI was separated from the other two time points (S3 Fig, Table B in S2 File). In order to directly plot the miRNA expression patterns in germinating rice seed, we normalized the TPM values through dividing each miRNA TPM by its corresponding maximum and reaching the abundance between 0 and 1. After normalization, one miRNA that was detected in at least one library with the normalized value lower than 0.5 (ratio >2 fold) was regarded as differentially expressed. A total of 178 differentially expressed miRNAs were obtained in germination initiation stage (Table C in S2 File), most (128 miRNAs) of which were enriched in the 24 HAI. Only four miRNAs were enriched in the dry seeds, especially for ptc-miR6478 and a novel miRNA OsmiR-201.

**Fig 5:**
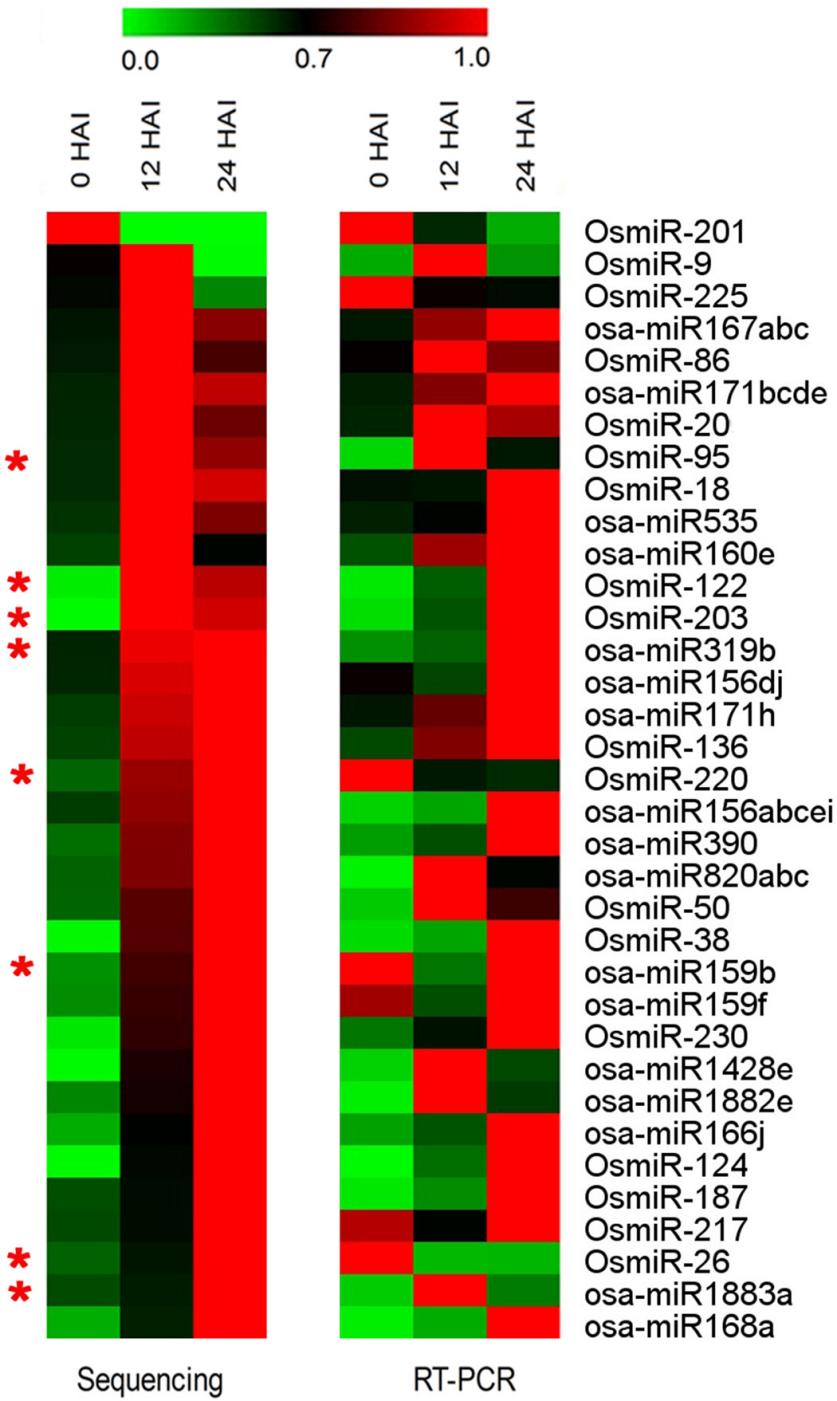
Heatmaps of sequencing data and stem-loop real-time RT-PCR. The bar represents the scale of the expression levels of miRNAs. The stars indicate inconsistent results between sequencing and RT-PCR.

To validate the miRNAs expression patterns obtained by deep sequencing, the 35 selected miRNAs were further verified by stem-loop real-time RT-PCR. The results showed that 27 miRNAs (about 77%) had expression patterns consistent to those in initial sequencing (Fig 5). However, some inconsistent results were also observed, such as OsmiR-220 and OsmiR-26, which were identified to be up-regulated remarkably with imbibition by sequencing but down-regulated by RT-PCR analysis, reflecting the dynamic change of the miRNAs expressions.

The intragenic miRNA, especially for the intronic miRNA, may be derived from a common precursor transcript and display coincidental expression pattern with the host gene [32,33]. We also investigated the expression relationship between miRNAs and the host genes inferred from the transcriptome dataset (GSE43780) [5]. However, neither the exonic nor intronic miRNAs had significant co-expression with their host genes, suggesting that these miRNAs were controlled by different promoters with their host genes (S4 Fig, S4 Table).

### Target Prediction and Function Analysis of Identified miRNAs

**Fig 6:**
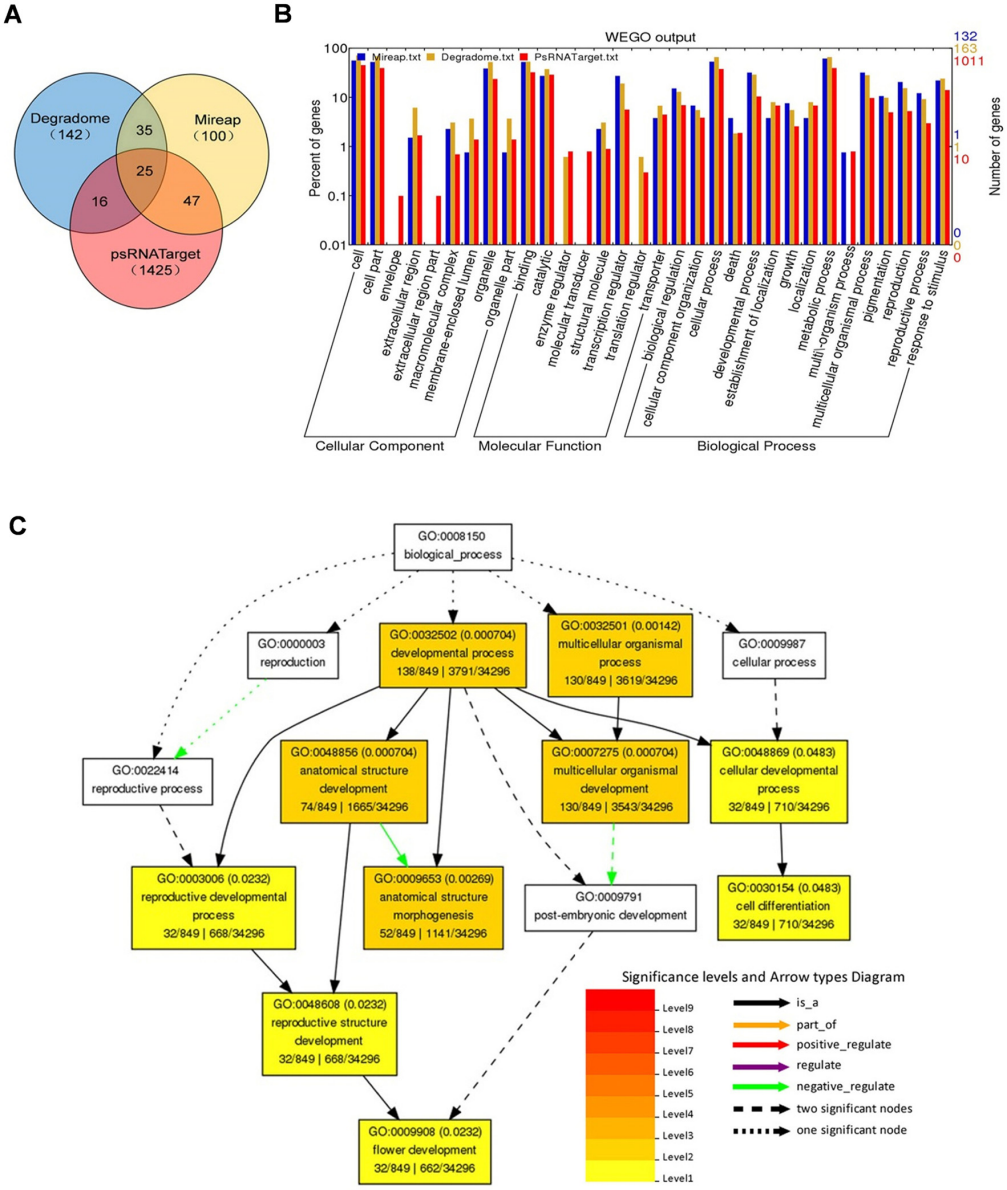
Gene ontology (GO) analysis of miRNAs’ target genes. **(A)** Venn diagram shows the overlapping relationship of the target prediction results among the three methods. **(B)** Categorization of miRNAs target genes with WEGO (http://wego.genomics.org.cn/cgi-bin/wego/index.pl). **(C)** GO enrichment of target genes using Agrigo (http://bioinfo.cau.edu.cn/agriGO/). The color scale shows the P-value cutoff levels for each biological process. The more statistically significant it is, the darker or redder the color is.

The target genes of all identified miRNAs were predicted using three classical methods: Mireap, degradome and psRNATarget analysis. After searching the rice genome dataset MSU7.0, 132, 163, 1011 target genes were obtained for 72, 133, and 242 miRNAs by the three methods, which were assembled into 207, 217, and 1508 miRNA-target pairs, respectively. Finally, a total of 1197 target genes for 259 miRNAs were found, which constituted 1784 miRNA-target pairs (S5 Table). Unexpectedly, only 25 pairs (21 genes targeted by 15 known miRNAs and one novel miRNA) were shared by all three prediction methods, and 123 (82 genes targeted by 26 known miRNAs and 24 novel miRNAs) pairs were shared by at least two methods (Fig 6A). Of the 21 common targets, most were conserved transcription factors for ancient miRNAs, including SQUAMOSA PROMOTER BINDING PROTEIN-LIKE (*SPL*s) protein coding genes targeted by miR156/miR529, *ARF*s targeted by miR160, *TCP*s targeted by miR319, and *MYB*s targeted by miR159.

All the putative target genes were categorized through gene ontology (GO) analysis. The results showed that these genes were involved in various molecular functions, i.e., binding, catalytic activity, and transcription regulation, and played roles in many biological processes, i.e., metabolic and cellular processes and signaling (Fig 6B). The Singular Enrichment Analysis (SEA) results of Agrigo software indicated that target genes related to the anatomical structure development (p = 2*10^−6^) and multicellular organismal development (P = 5.7*10^−6^) were significantly enriched in the biological process category (Fig 6C). In contrast, no GO term was enriched for the categories of cellular component and molecular function.

Furthermore, the expression profiles of target genes were extracted from the transcriptome dataset (GSE43780) [5]. In general, the mRNA expression profiles were negatively correlated with their corresponding miRNAs (Table A in S3 File). Of the 178 differentially expressed miRNAs, 28 (16 known and 12 novel) miRNAs with sequencing reads over 100 were significantly expressed opposite to their targets (Folds > 2, Correlation index < -0.5, Table B in S3 File). These targets included 39 genes encoding proteins for major and minor metabolism, protein degradation and post-translational modification, transportation, and transcriptional regulation, which participated in cellular, metabolic, growth, developmental, and other biological processes.

### Verification of miRNAs-Induced Cleavage by 5’-RACE

To validate the authentic cleavage event of the known or novel miRNAs in the germinating rice seed, some inversely expressed miRNA-target pairs were selected to verify the cleavage site through 5ʹ-RACE.

**Fig 7:**
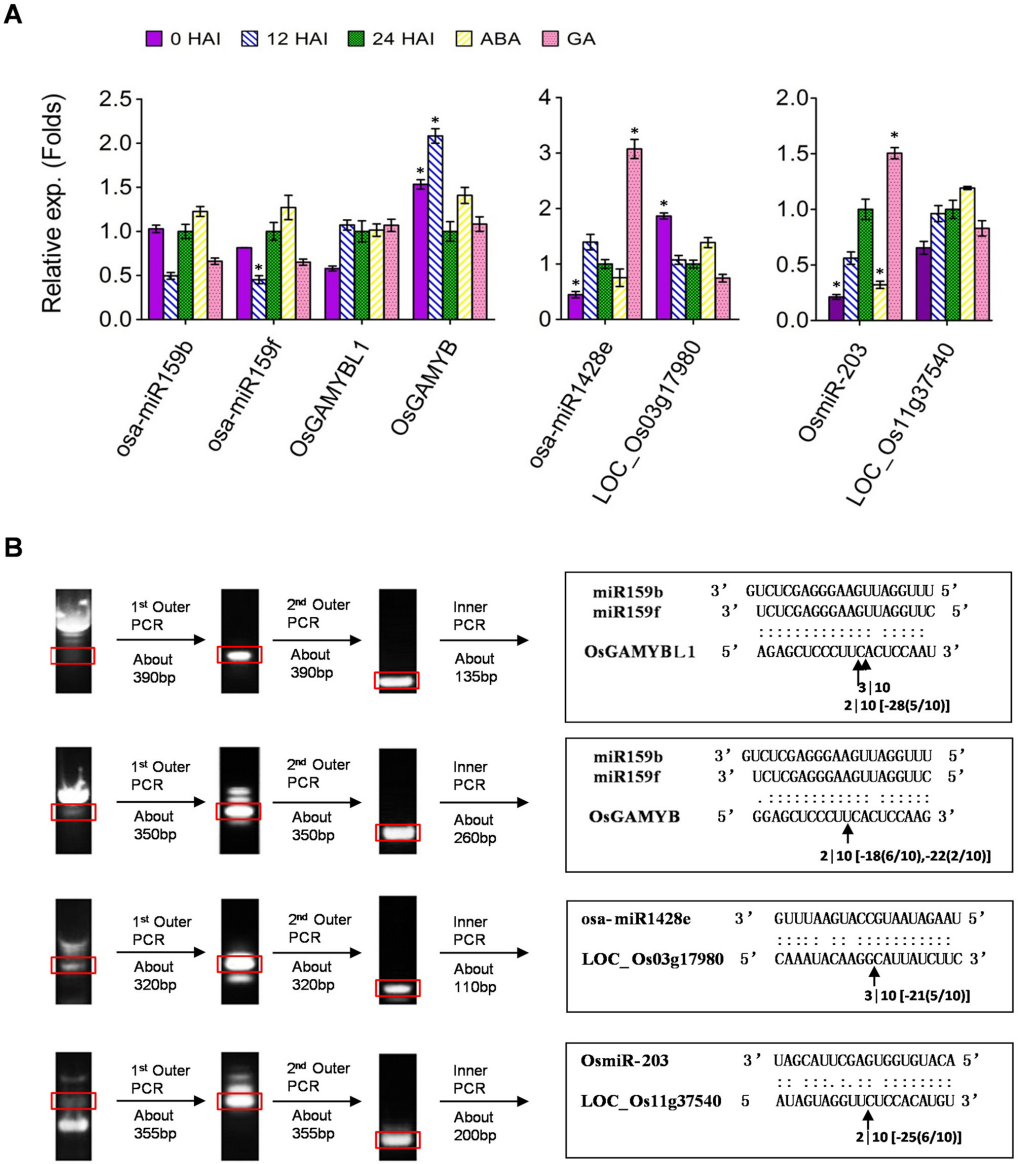
Real-time RT-PCR and 5’-RACE analysis. **(A)** Real-time RT-PCR analysis of the relative expression of osa-miR159b, osa-miR159f, osa-miR1428e and OsmiR203 and their corresponding targets in embryos of 0, 12, and 24 HAI and ABA- or GA- treated seeds. The data represent the mean values ± SD of three replicates. The rice 18S rRNA fragment was amplified as internal control. Asterisks (* p < 0.01, Student’s t-test) represent significant differences from the expression level in 24 HAI seed embryos. **(B)** Validation of target mRNA cleavage sites by RNA ligase mediated 5ʹ -RACE. The rectangles on the gel show the bands of nested PCR products. The arrows indicate the cleavage sites, the numbers under the arrows show the frequency of RACE clones sequenced, and the numbers in the brackets show the position and frequency of cleavage sites downstream of the canonical sites.

GAMYB is well documented as the gibberellin (GA) signaling regulator in cereal aleurone cells [34]. The transcriptome data of germinating rice seeds indicated that both *OsGAMYBL1* (*LOC_Os06g40330*) and *OsGAMYB* (*LOC_Os01g59660*) were remarkably down-regulated in the first 24 HAI. Our RT-PCR results showed that the mRNA level of *OsGAMYBL1* was constant while the level of *OsGAMYB* decreased in the first 24 h of imbibition (Fig 7A). ABA and GA are two vital phytohormones in regulating seed germination. We detected ABA and GA responses of the selected miRNAs and their targets in this study. RT-PCR results showed that both miR159 and *GAMYB*s were insensitive to ABA and GA in rice embryos. RNA ligase-mediated 5ʹ-RACE indicated that *OsGAMYBL1* and *OsGAMYB* produced PCR products with canonical cleavage sites of miR159 (Fig 7B). Unexpectedly, we also found some fragments mapped down-stream of the canonical cleavage sites, which reflected the existence of RNA degradation after cleavage, and hence the post-transcriptional modification.

Real-time RT-PCR results indicated that rice special miRNA osa-miR1428e-3p was increased during seed imbibition and induced greatly by GA. Inversely, its target *OSK3* (a carbon catabolite depressing protein kinase encoded by *LOC_Os03g17980*) was decreased during seed imbibition and insensitive to GA and ABA. OsmiR-203 was induced by GA as well, and its target *LOC_Os11g37540* (encoding a protein phosphatase 2C family protein) displayed consistent expression under all detected conditions (Fig 7A). The 5ʹ-RACE also detected the canonical cleavage sites on the *OSK3* and *LOC_Os11g37540* for osa-miR1428e-3p and OsmiR-203, while most clones carried fragments matched to the downstream sequences, suggesting that mRNA fragments induced by miRNA-mediated cleavage were easily degraded from the 5ʹ terminal (Fig 7B).

## Discussion

### Specific miRNA Profiles and Expression Patterns in Dry and Imbibed Rice Seeds

The rice dataset of the miRBase (version 21) has recorded 688 precursors for mature miRNAs from 332 families. However, we identified only 83 precursors processed for 59 known miRNAs belonging to 42 families in rice seeds; this ratio is relatively lower than that in leaf, root and pollen [16]. Moreover, the HCL analysis and PCA indicated that the known miRNA expression profiles in the embryo of rice seeds were significantly separated from that of the leaf, root, callus and developing pollen (Fig 4). Particularly, some known miRNAs, i.e., osa-miR1876 and osa-miR5150, were abundantly expressed in rice seed but were not detected in other tissues, and osa-miR159f, osa-miR166l, and osa-miR319b were predominantly expressed in the seed, which suggested the pivotal regulatory roles of these miRNAs in seed germination. The size distribution of miRNAs has been revealed to be tissue-specific in Arabidopsis and rice [16,35]. Most of the conserved miRNAs are short sRNAs with size ranging from 20 to 22 nt [7]. The 24-nt miRNAs are involved in DNA methylation or histone modifications leading to transcriptional gene silencing and chromatin remodeling [36,37]. In imbibed seeds, many 24-nt miRNAs with low abundance and fluctuating expression patterns were identified (S2 and S3 Tables), and most of them were novel. From the above analysis, we concluded that the unique miRNAs composition and expression profiles existed in dry and imbibed rice seeds.

The composition of miRNAs in germinating rice seed was relatively stable as 277 of 289 identified miRNAs were shared by three libraries (Fig 1), whereas their expression pattern varied greatly. Altogether, 178 differentially expressed miRNAs were identified, and only few miRNAs had higher TPM values in dry seeds, such as ptc-miR6478 and OsmiR-201 (Table C in S2 File). However, the miRNAs expression patterns between 12 and 24 HAI were similar. HCL analysis showed that the expression profile of miRNAs in 0 HAI seed embryos was separated from that of the other two time points (S4 Fig). The drastic turnover of the miRNAs profile from dry seeds to imbibed seeds suggested that miRNA-mediated regulatory metabolisms were rapidly activated after imbibition.

### Roles of miRNAs in Initiation of Rice Seed Germination

Since protein translation from the stored long-lived mRNAs is indispensable for seed germination, the miRNA-mediated post-transcription regulation can play key roles in the mRNA selective translation and protein turnover during this process [38,39]. To comprehensively explore the possible roles of identified miRNAs during seed imbibition, two widely accepted softwares, Mireap and PsRNATarget, were used to predict the miRNA targets. According to the miRNA-target sequence complementation, it is relatively easy to predict the plant miRNA targets with the reference genomic or transcriptomic sequence data [23]. However, the computational prediction is challenged by a high level of false positives that exist in miRNA-target pairing [40]. Degradome sequencing is a recently established, efficient strategy to identify miRNA targets on a large scale [22,41]. In this study, degradome analysis was also used for targets prediction based on the published datasets. In total, 1197 target genes involved in various functions for 259 miRNAs were obtained by three prediction methods. However, the remaining 30 miRNAs were failed to find potential targets. These miRNAs might regulate gene expression through non-stringent complementary paring, such as translational repression [42,43], or guide the non-coding locus cleavage and produce phasi/tasiRNA [8]. GO enrichment results indicated that target genes involved in anatomical structure development and multicellular organismal development, i.e., wall-associated protein kinases (*OsWAK*s), expansin precursors, and *OsSPL*s, were over-represented. This is consistent with the development process of germination initiation, during which many cell structures need to be repaired or synthesized *de novo*. Unexpectedly, only 25 miRNA-target pairs overlapped in the three methods of analysis. The ultra-low overlap among these target prediction methods reflected a difference in the parameter setting or prediction principle. This is also strongly suggestive of the predicted targets which need to be confirmed experimentally since the result varied with different prediction methods.

Transcription factors such as *TCP*s, *ARF*s and *SPL*s have been confirmed to be easily targeted by conserved miRNAs. By repressing *TCP*s, miR319 played a central role in coordinating multiple miRNAs (i.e., miR396 and miR164) and phytohormones (including auxin, ABA and GA) pathways to control lateral organ development [44,45]. Auxin alone is not generally considered as a typical seed germination hormone, but it was revealed to interact with ABA through *ARF10* targeted by miR160 and to be involved in seed germination and post-germination control [11]. The auxin signal affects rice root development through regulation of miR156-SPLs [46]. SPL proteins have also been revealed to restrain the transition from post-germination to autotrophic growth and affect seedling establishment [31,47]. In this study, osa-miR319 and osa-miR156abcdejl showed high abundance, but miR160abe was relatively low-expressed in all three libraries. Since the complex biological process of seed germination is regulated by a great interaction network of phytohormones, these conserved miRNAs are devoted to the germination initiation and the following seedling establishment through targeting the related signaling regulation factors. However, until now, the mechanisms of miRNAs-mediated phytohormone crosstalk involving in seed germination are largely unknown.

The first 24 HAI is critical for initiation of rice seed germination, during which RNA transcription, metabolism resumption and cellular repair are highly activated [19,38,48,49]. According to the published transcriptome data, 28 miRNAs were found to be expressed remarkably opposite to their targeted genes (Table B in S3 File), including conserved miRNAs, i.e., miR169ijk targeted nuclear factors, miR390 targeted ATP binding proteins, and miR166e targeted SGT1 protein, which are known to participate in plant development and disease resistance [50]. Novel miRNA-target pairs provide useful information to explore the new functions of miRNAs in rice seed germination. For example, OsmiR-129 sharply increased during seed imbibition, it might reduce the phytic acid synthesis in rice seeds by targeting a multi-drug resistance-associated ABC transporter and release micronutrients and organic phosphorus for seed germination [51]. Notably, among those inversely expressed miRNA-target pairs, several miRNAs were found to target genes with unknown function, i.e., OsmiR-100 (LOC_Os07g07030), OsmiR-217 (LOC_Os07g08669), OsmiR-232 (LOC_Os05g06910). It is worth in-depth investigation on the roles of those miRNA-target pairs in the seed germination regulation.

The expression level of miRNAs are not only inversely but sometimes also positively correlated with their target genes for feedback regulation or other interference factors [16]. In this study, many differentially expressed miRNAs were identified to be positively correlated with their target genes, such as OsmiR-201. OsmiR-201 is highly expressed in dry seeds but the expression decreases rapidly during imbibition. Three potential targets of OsmiR-201, LOC_Os05g50660 (encoding a PX domain containing protein), LOC_Os01g69940 (encoding an F-box domain containing protein), and LOC_Os01g62800 (encoding a methyltransferase) decrease during seed imbibition. Exploring whether the feedback regulations exist in these kinds of miRNA-target pairs will help to understand the miRNA-mediated regulations from a new angle.

### MicroRNA Mediates the Crosstalk between ABA and GA in Controlling Rice Seed Germination

ABA is well documented as a seed dormancy maintainer that functions antagonistically to GA. MicroRNA159 has been reported extensively to be involved in the crosstalk between ABA and GA through cleavage of *GAMYB*-like genes in leaves, flowers, seed maturation and germination process [34,52,53]. In Arabidopsis seeds, exogenous ABA results in the accumulation of miR159 that participates in the regulation of seed germination by targeting MYB33 and MYB101, two positive regulators of ABA response [53]. This negative feedback might contribute to the developmental switch from seed dormancy to germination in Arabidopsis [54]. In rice, expression of miR159 was detected in various organs except in seeds. However, its target genes, *OsGAMYBL1* and *OsGAMYB*, which regulate almost all GA-regulated genes in aleuronic cells, are highly expressed in the seed aleurone layer and promote storage hydrolysis during seed germination [34]. The expression level of miR159 is found to be very low in the embryo of non-dormant maize seeds [55]. Unexpectedly, in this study, we identified abundant miR159, especially miR159f, in the embryos of germinating rice seeds. In addition, we observed that miR519 was insensitive to ABA or GA. Thus, the plentiful miR159 in rice embryo cannot be induced by ABA as it is in Arabidopsis. RACE analysis verified that *OsGAMYBL1* and *OsGAMYB* were both cleaved by miR159 at the canonical positions. Meanwhile, *OsGAMYB* (the ortholog of Arabidopsis MYB33) was detected to be down-regulated by RT-PCR and microarray analysis in the embryos of germinating rice seeds (S3 File and Fig 7A). It is of interest to reveal the distinctive expression patterns of osa-miR159 and the role of osa-miR159 mediated crosstalk between ABA and GA in the embryo of germinating rice seed in future studies.

Two other targets potentially involved in the ABA signaling were also verified by 5ʹ-RACE. One is *OSK3*, targeted by rice known miRNA osa-miR1428e-3p. *OSK3* has been identified as an important gene for starch accumulation in the early stages of rice endosperm development [56]. During seed germination, most proteins related to rice endosperm development are rapidly degraded [19,57]. Similarly, *OSK3* was detected to decrease drastically by RT-PCR in seed imbibition. *OSK3* belongs to the sucrose non-fermenting related kinase 1 (*SnRK1*) family, which is well documented as a central component of the regulatory response to glucose starvation and participates in ABA signaling pathways [58,59]. However, RT-PCR results showed that *OSK3* was insensitive to ABA or GA in the embryo of germinating rice seed, suggesting that the function of *OSK3* might be regulated by ABA at the post-translational level. In contrast, osa-miR1428e-3p was induced significantly by GA, implying that GA displayed antagonistic role to ABA through up-regulating the inhibitor of the positive regulator in ABA signaling. Gene *LOC_Os11g37540* encoding a PP2C domain containing protein was predicted to be targeted by a novel miRNA OsmiR-203. Rice has 90 PP2C proteins, and most of them participate in ABA signaling by interacting with SnRKs and suppressing SnRKs activation through dephosphorylation of Ser/Thr residues in the activation loop [58,60]. However, the RT-PCR results indicated that *LOC_Os11g37540* was insensitive to ABA or GA, and OsmiR-203 was slightly increased by GA but inhibited by ABA. These observations reflect the complex crosstalk between ABA and GA mediated by miRNAs in controlling rice seed germination.

## Supporting Information

S1 Fig Abundance of conserved miRNAs in three miRNA datasets of 0, 12 and 24 HAI rice seed embryos. (TIF)

S2 Fig Verification of 35 identified miRNAs by stem-loop RT-PCR. Lanes 3–19 are the 17 known rice miRNAs: osa-miR156abcei, osa-miR156dj, osa-miR160e, osa-miR166j, osa-miR167abc, osa-miR168a, osa-miR171bcde, osa-miR171h, osa-miR319b, osa-miR390, osa-miR535, osa-miR820abc, osa-miR1428e, osa-miR1862abc, osa-miR1882e, osa-miR1883a, and osa-miR5150. Lanes 20–37 are the 18 novel miRNAs OsmiR-9, OsmiR-18, OsmiR-20, OsmiR-26, OsmiR-38, OsmiR-50, OsmiR-86, OsmiR-95, OsmiR-122, OsmiR-124, OsmiR-136, OsmiR-187, OsmiR-201, OsmiR-203, OsmiR-217, OsmiR-220, OsmiR-225, and OsmiR-230. Lane M1, 100 bp DNA ladder size marker. Lane M2, 50 bp DNA size marker.
(TIF)

S3 Fig Hierarchical clustering analyses of all miRNAs in 0, 12 and 24 HAI rice seeds embryos. The bar represents the scale of the miRNAs expression levels. The detailed expression information is listed in the S4B Table.
(TIF)

S4 Fig Correlation of expression of intronic or exonic miRNAs and their corresponding host genes. The bar represents the scale of the miRNAs expression levels, TPM values of miRNAs and GCRMA values of mRNAs were normalized between 0 and 1. The detailed information is listed in S5 Table.
(TIF)

S1 File Characteristics of the miRNA precursor candidates. Minimal folding free energy **(Figure A)**, size **(Figure B)** and GC% content **(Figure C)** distributions of the miRNA precursor candidates.
(TIF)

S2 File Expression analysis of identified miRNAs. Expression profiling of known miRNAs in seven libraries (**Table A**). Normalizating the expression of all miRNAs by Log2TPM for heatmap (**Table B**). All 178 differentially expressed miRNAs (**Table C**).
(XLSX)

S3 File Expression profiles of miRNAs and their corresponding targets. Expression profiles of all predicted miRNA-target pairs (**Table A**). Oppositely expressed miRNA-target pairs (**Table B**).
(XLSX)

S1 Table Primers used in this study. (XLSX)

S2 Table All identified known miRNAs. (XLSX)

S3 Table All predicted novel miRNAs. (XLSX)

S4 Table Expression profiles of intronic or exonic miRNAs and their host genes. (XLSX)

S5 Table Target gene prediction for all identified miRNAs using three methods. (XLSX)
