# Supplementary figures and images for: Genome-Wide Dissection of the MicroRNA Expression Profile in Rice Embryo during Early Stages of Seed Germination
Source: PLoS One. 2015 Dec 17;10(12):e0145424. doi: 10.1371/journal.pone.0145424 (PMC4683037; doi:10.1371/journal.pone.0145424)

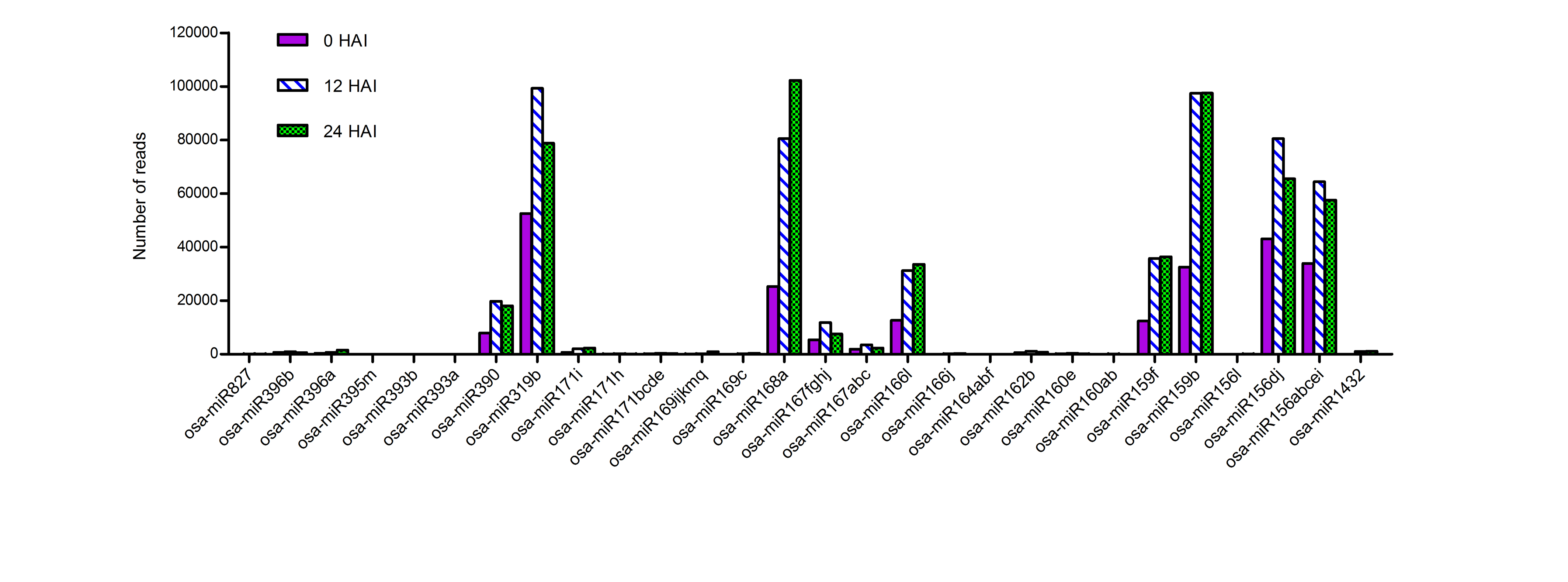

Supplement: S1 Fig — (TIF) [file pone.0145424.s001.tif]

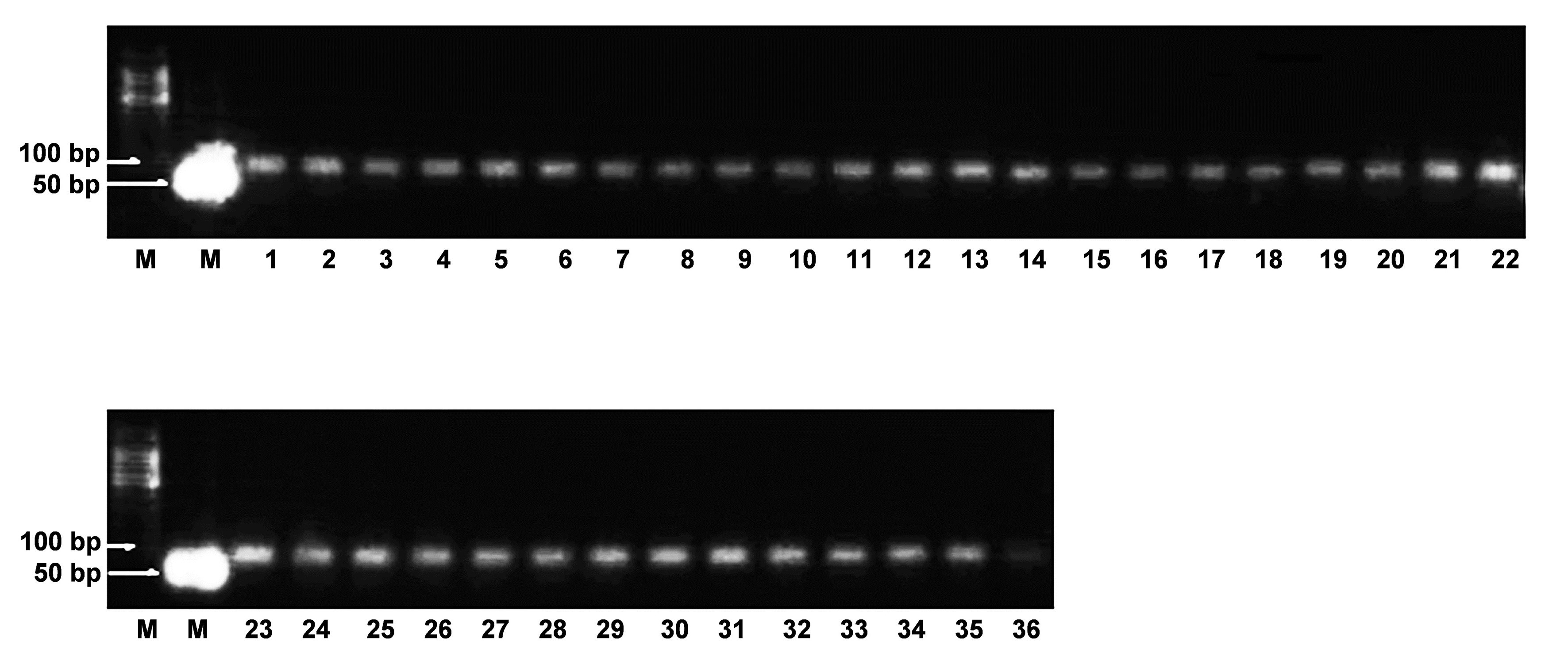

Supplement: S2 Fig — Lanes 3–19 are the 17 known rice miRNAs: osa-miR156abcei, osa-miR156dj, osa-miR160e, osa-miR166j, osa-miR167abc, osa-miR168a, osa-miR171bcde, osa-miR171h, osa-miR319b, osa-miR390, osa-miR535, osa-miR820abc, osa-miR1428e, osa-miR1862abc, osa-miR1882e, osa-miR1883a, and osa-miR5150. Lanes 20–37 are the 18 novel miRNAs OsmiR-9, OsmiR-18, OsmiR-20, OsmiR-26, OsmiR-38, OsmiR-50, OsmiR-86, OsmiR-95, OsmiR-122, OsmiR-124, OsmiR-136, OsmiR-187, OsmiR-201, OsmiR-203, OsmiR-217, OsmiR-220, OsmiR-225, and OsmiR-230. Lane M1, 100 bp DNA ladder size marker. Lane M2, 50 bp DNA size marker. (TIF) [file pone.0145424.s002.tif]

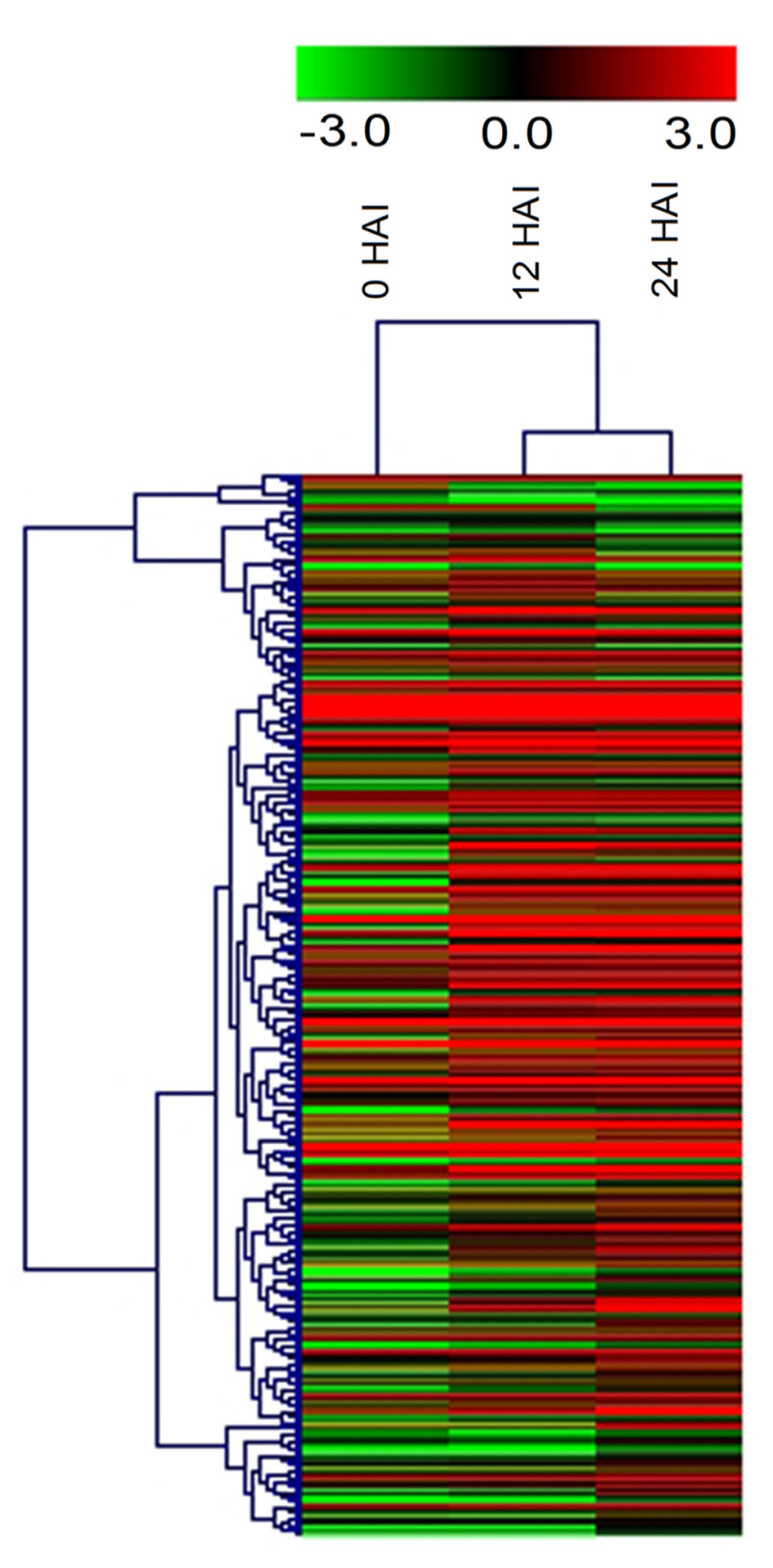

Supplement: S3 Fig — The bar represents the scale of the miRNAs expression levels. The detailed expression information is listed in the S4B Table. (TIF) [file pone.0145424.s003.tif]

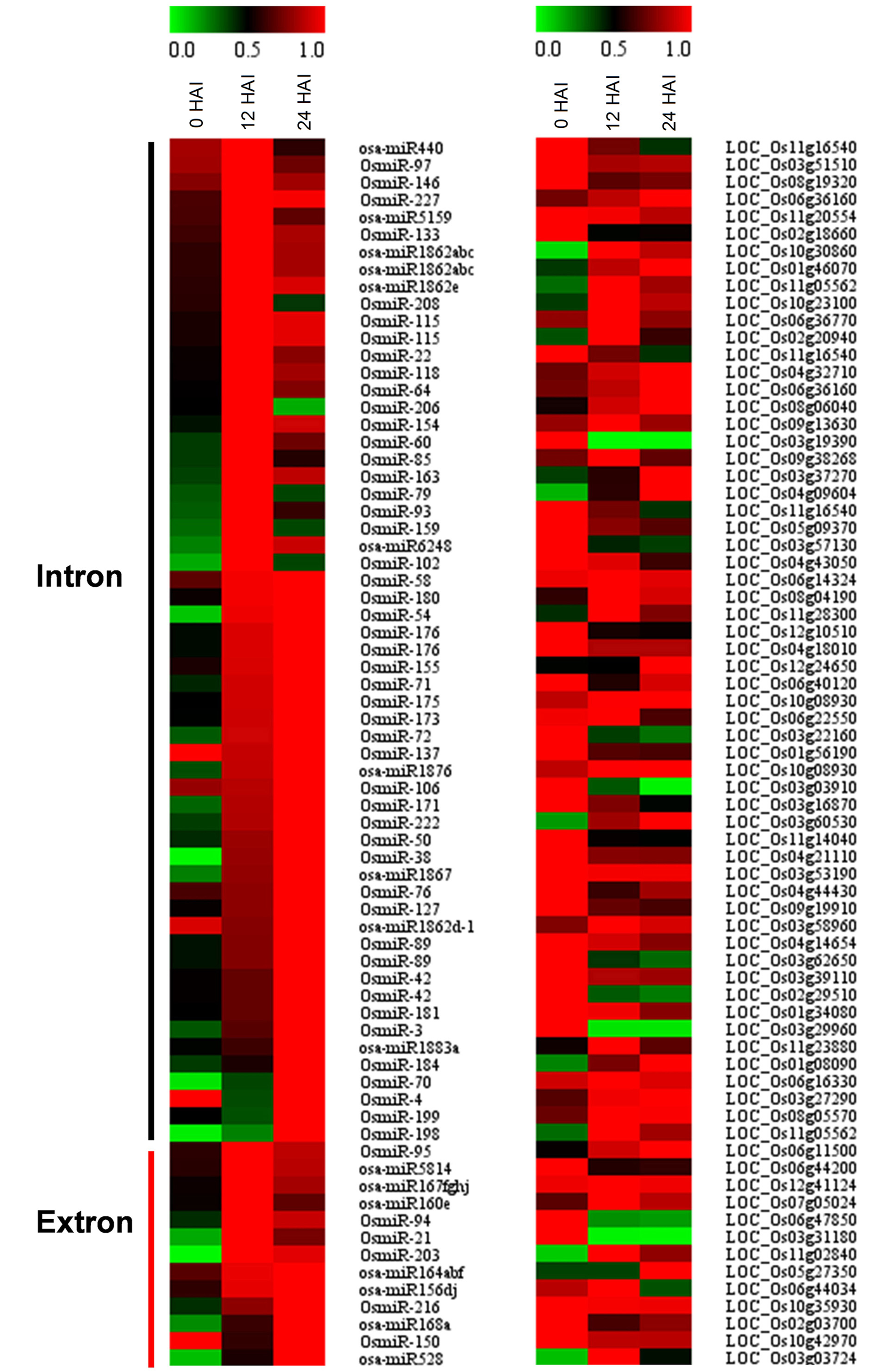

Supplement: S4 Fig — The bar represents the scale of the miRNAs expression levels, TPM values of miRNAs and GCRMA values of mRNAs were normalized between 0 and 1. The detailed information is listed in S5 Table. (TIF) [file pone.0145424.s004.tif]

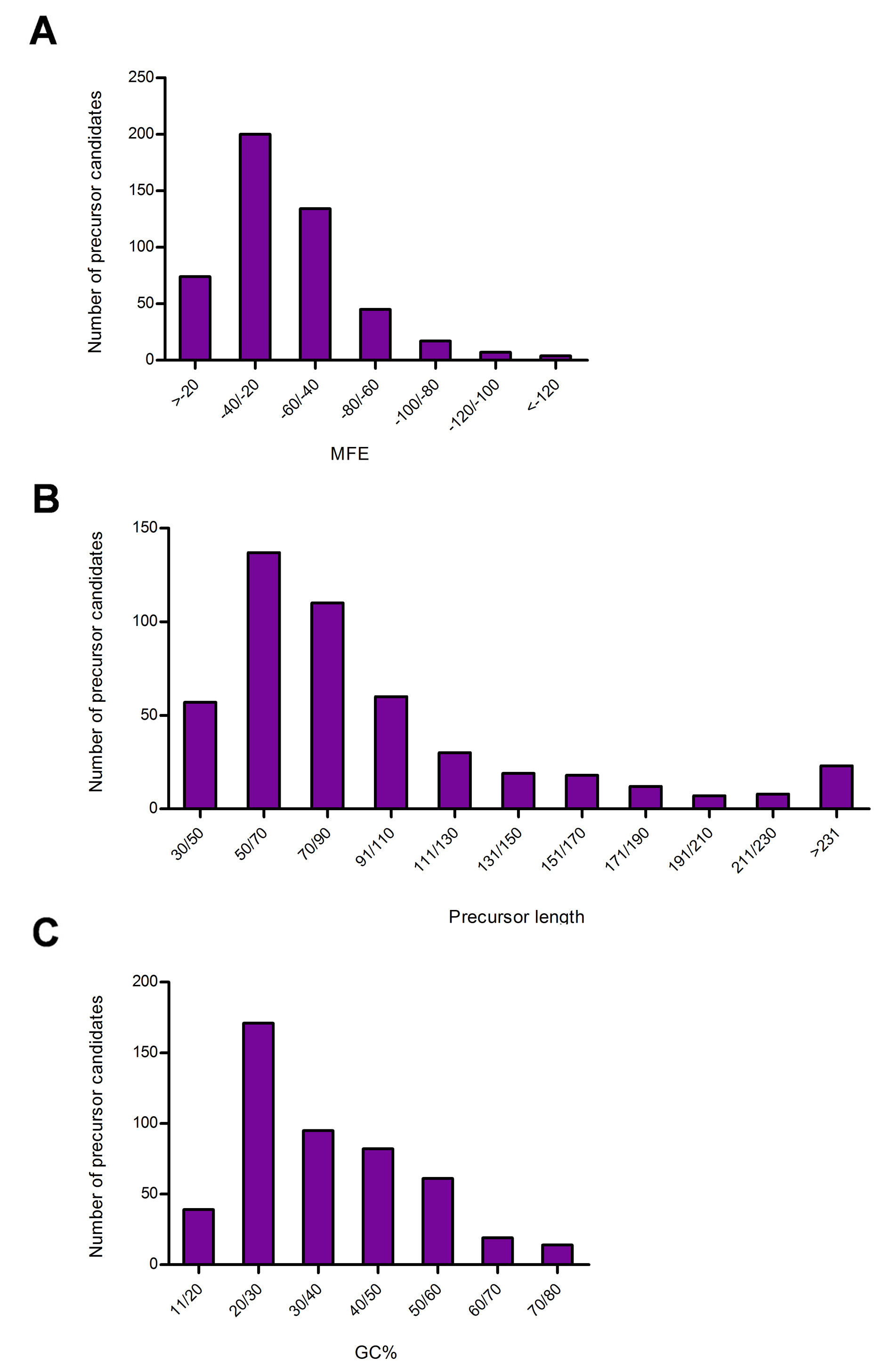

Supplement: S1 File — Minimal folding free energy (Figure A), size (Figure B) and GC% content (Figure C) distributions of the miRNA precursor candidates. (TIF) [file pone.0145424.s005.tif]
